# Evaluation of the maximum tongue and lip pressure in individuals with Class I, II, or III Angle malocclusions and different facial types

**DOI:** 10.1590/2317-1782/20232022102

**Published:** 2023-07-10

**Authors:** Fernanda Alvarenga Guimarães Martins, Andréa Rodrigues Motta, Leniana Santos Neves, Renata Maria Moreira Moraes Furlan

**Affiliations:** 1 Programa de Pós-graduação em Ciências Fonoaudiológicas, Universidade Federal de Minas Gerais – UFMG - Belo Horizonte (MG), Brasil.; 2 Departamento de Fonoaudiologia, Universidade Federal de Minas Gerais – UFMG - Belo Horizonte (MG), Brasil.; 3 Departamento de Odontologia Restauradora, Universidade Federal de Minas Gerais – UFMG - Belo Horizonte (MG), Brasil.

**Keywords:** Muscle Strength, Lip, Malocclusion, Angle Class I, Malocclusion, Angle Class II, Malocclusion, Angle Class III, Força Muscular, Lábio, Má Oclusão Classe I de Angle, Má Oclusão Classe II de Angle, Má Oclusão Classe III de Angle

## Abstract

**Purpose:**

To compare the maximum anterior and posterior tongue pressure, tongue endurance, and lip pressure in Class I, II, and III malocclusions and different facial types.

**Methods:**

A cross-sectional observational analytical study was carried out in 55 individuals (29 men and 26 women) aged between 18 and 55 years. The participants were divided into groups according to Angle malocclusion (Class I, II, and III) and facial type. The maximum anterior and posterior tongue pressure, tongue endurance, and maximum lip pressure were measured using the IOPI (Iowa Oral Performance Instrument). To determine the facial type, the cephalometric analysis was accomplished using Ricketts VERT analysis as a reference.

**Results:**

There was no statistically significant difference when comparing the maximum pressure of the anterior and posterior regions of the tongue, the maximum pressure of the lips, or the endurance of the tongue in the different Angle malocclusion types. Maximum posterior tongue pressure was lower in vertical individuals than in mesofacial individuals.

**Conclusion:**

Tongue and lips pressure, as well as tongue endurance in adults was not associated with the type of malocclusion. However, there is an association between facial type and the posterior pressure of the tongue.

## RESUMO

**Objetivo:**

comparar a pressão máxima anterior e posterior da língua, a resistência da língua e a pressão labial em indivíduos com más oclusões Classe I, II e III e diferentes tipos faciais.

**Método:**

foi realizado um estudo analítico observacional transversal em 55 indivíduos (29 homens e 26 mulheres) com idades entre 18 e 55 anos. Os participantes foram divididos em grupos de acordo com a classificação de Angle para má oclusão (Classe I, II e III) e tipo facial. A pressão máxima anterior e posterior da língua, a resistência da língua e a pressão máxima dos lábios foram medidas usando o IOPI (Iowa Oral Performance Instrument). Para determinar o tipo facial, a análise cefalométrica foi realizada utilizando como referência a análise Ricketts VERT.

**Resultados:**

não houve diferença estatisticamente significativa ao comparar a pressão máxima das regiões anterior e posterior da língua, a pressão máxima dos lábios ou a resistência da língua nos diferentes tipos de má oclusão. A pressão máxima posterior da língua foi menor em indivíduos com tipo facial vertical do que nos indivíduos mesofaciais.

**Conclusão:**

a pressão de língua e lábios, assim como a resistência de língua em adultos não foi associada ao tipo de má oclusão. No entanto, existe uma associação entre o tipo facial e a pressão posterior da língua.

## INTRODUCTION

The tongue is composed of intrinsic and extrinsic muscles organized in complex and intricate arrays of fibers that contract synergistically to produce different physiological movements during chewing, swallowing, and speech^(1)^. In order to understand tongue physiology, mainly during swallowing, some studies reported on the tongue strength in normal adults^(2,3)^. In addition, they also reported on the impact of reduced tongue strength on swallowing function^(4)^, and the benefits of tongue strengthening exercises in improving swallowing^(5)^.

In the early 1990s, new tools to measure the pressure generated by the tongue were developed, offering speech therapists and dentists a complementary means for measuring the pressure and endurance of the tongue. Later, these instruments were adapted to assess the pressure exerted by the lips^(6)^. Researches were carried out to establish normative values ​​for orofacial strength and endurance and to investigate the possibility of influences of age^(4)^, sex^(6)^, and clinical conditions such as mouth-breathing^(7)^, obstructive sleep apnea^(8)^, and temporomandibular disorders^(9)^. However, few studies have been conducted on the influence of malocclusion on the force or pressure exerted by the tongue and lips^(10-16)^.

Tomes (1873) stated that opposing forces exerted by the lips and cheeks on one side and by the tongue on the other are determinants of the dental position^(17)^. This suggests the existence of a relationship between the strength of the musculature and the presence of malocclusion. However, the literature is contradictory on this topic. Silva et al.^(11)^ compared tongue strength between adults with Class II and Class III dentofacial deformities and between them and controls with normal dentofacial relation and did not find difference, neither in anterior nor in posterior tongue strength. Kuwajima et al.^(16)^ found no difference between maximum tongue pressure in individuals (children and adults) with different Angle classifications. Partal and Aksu^(12)^ measured tongue and lip pressure of children with Class II dental malocclusion and did not find a difference between them and those with Class I dental malocclusion. On the other side, other authors^(13,15)^ found an association between tongue pressure and maxillofacial morphology in children and suggest a relationship between tongue position and its force.

Because there are few studies associating orofacial pressures and maxillofacial morphology^(11,13-15)^, and even fewer associating those pressures with non-skeletal malocclusion^(10,12,16)^, these relationships remain unclear. In addition, less attention has been paid to the relationship between tongue endurance and malocclusion, as just one study on this topic was found^(14)^.

Another factor that may influence orofacial strength is the facial type. Berwig et al.^(18)^ analyzed tongue and lip position in different facial types and found a trend in vertical individuals (those with narrower transverse dimensions) of presenting half-open or open lips, and lower tongue posture. The vertical growth of the face, mainly in the lower third, makes it difficult for the lower lip to reach in the direction of the upper lip, and the mandible elevator muscles are more stretched out and less powerful, resulting in a lowered mandibular position, which also compromises the proper usual lips position^(18)^, and consequently its force^(7)^. Besides, in the vertical growth pattern, due to the increase of the lower third of the face it is difficult for the individual to keep tongue in contact with the palate^(18)^. The lowered position of the tongue, in turn, can cause a decrease in its strength^(7)^.

This study aimed to compare tongue pressure, lip pressure, and tongue endurance between individuals with Class I, II, and III malocclusions and between different facial types. We hypothesize that tongue pressure, tongue endurance and lip pressure are reduced in Class II and Class III individuals compared to Class I individuals, and in vertical compared to horizontal-face individuals, as in these conditions the tongue usually stays in a lowered habitual position and there is lack of lip seal.

## METHODS

An observational cross-sectional study was conducted after obtaining approval from the Research Ethics Committee of Universidade Federal de Minas Gerais (CAAE 96220318.3.0000.5149) under the number 2.912.714. The study was performed in accordance with the ethical standards of the 1964 Declaration of Helsinki and its later amendments. All participants provided written informed consent for participation.

### Sample

The study included 55 healthy individuals between 18 and 55 years of age. For the sample calculation, the data obtained from measuring tongue pressure of 34 adults in a recent study was used as a reference^(14)^. Thus, considering a power of 80% and a significance level of 5%, the number of individuals for the present study should be at least 51 individuals.

The participants were recruited in Orthodontic Clinics among individuals who were going to start orthodontic treatment. The sample was divided into three groups according to the occlusion classification: Group 1 included individuals with Angle Class I malocclusion, Group 2 included individuals with Angle Class II malocclusion, and Group III individuals with Angle Class III malocclusion.

Inclusion criteria were as follows: 1) 18 years or older, 2) presence of all permanent first molar teeth, 3) cephalometric radiography present in the orthodontic documentation and 4) no cognitive, visual, or auditory impairments that could interfere with the test or invasive developmental disorders. Individuals who had already undergone previous orofacial myofunctional treatment that included tongue exercises, individuals using orthodontic appliances installed on the palate, those who presented with anterior and/or posterior open bite, or altered lingual frenulum were excluded.

Dental occlusion evaluation was performed by a dentist, according to Angle^(19)^ classification. The evaluation of the lingual frenulum was performed using the tongue frenulum evaluation protocol^(20)^.

### Facial type classification

For the classification of the facial type, Ricketts VERT analysis was used^(21)^. The VERT index was obtained from five cephalometric measurements: lower face height, facial depth, facial axis angle, mandibular plane angle, and mandibular arch.

To obtain the VERT index, the Radiomemory® software, version Radiocef Studio 3 (3.0.6.100) (Belo Horizonte, Minas Gerais, Brazil), was used. Each lateral cephalometric radiograph was calibrated before starting the analysis, which consisted of marking 65 cephalometric points. Afterwards, the software provided the measurement of studied variables and automatically calculated the VERT value. When VERT values ranged between -0.5 and 0.5, individuals were classified as mesofacial. Participants with values ​​greater than 0.5 were classified as horizontal, and values ​​less than -0.5 indicated vertical individuals. All procedures were performed by the same examiner.

Casual errors in the cephalometric analyses were evaluated using Dahlberg’s formula on 11 randomly selected cephalograms (which corresponded to approximately 20% of the sample), with a 4-week interval between measurements^(22)^. The paired *t*-test was used to assess the systematic error with a significance level of 5%. Measurements with a variation of up to 1 mm for linear quantities and 1.5º for angular quantities were considered acceptable^(23)^ (Chart 1).

**Chart 1:** Systematic and casual (Dahlberg) intra-examiner errors

Variables	Measurement 1 (n=11)		Measurement 2 (n=11)		p-value*	Dahlberg’s method error
	Mean	SD	Mean	SD		
Facial axis angle (º)	91.73	6.37	91.04	4.66	0.5991	2.8796
Facial depth (º)	88.66	3.62	89.02	3.30	0.7516	2.5503
Mandibular plane angle (º)	25.54	7.43	23.48	7.18	0.1742	3.4708
Lower face height (º)	41.66	3.72	41.70	4.31	0.9508	1.4783
Mandibular arch (º)	40.22	12.87	40.95	7.51	0.8483	8.3417
VERT index (º)	0.67	1.38	0.73	1.06	0.8141	0.5743

* Paired t-test

**Caption:** SD = standard deviation.

### Tongue and Lip pressure and tongue endurance measurements

**Figure 1:**
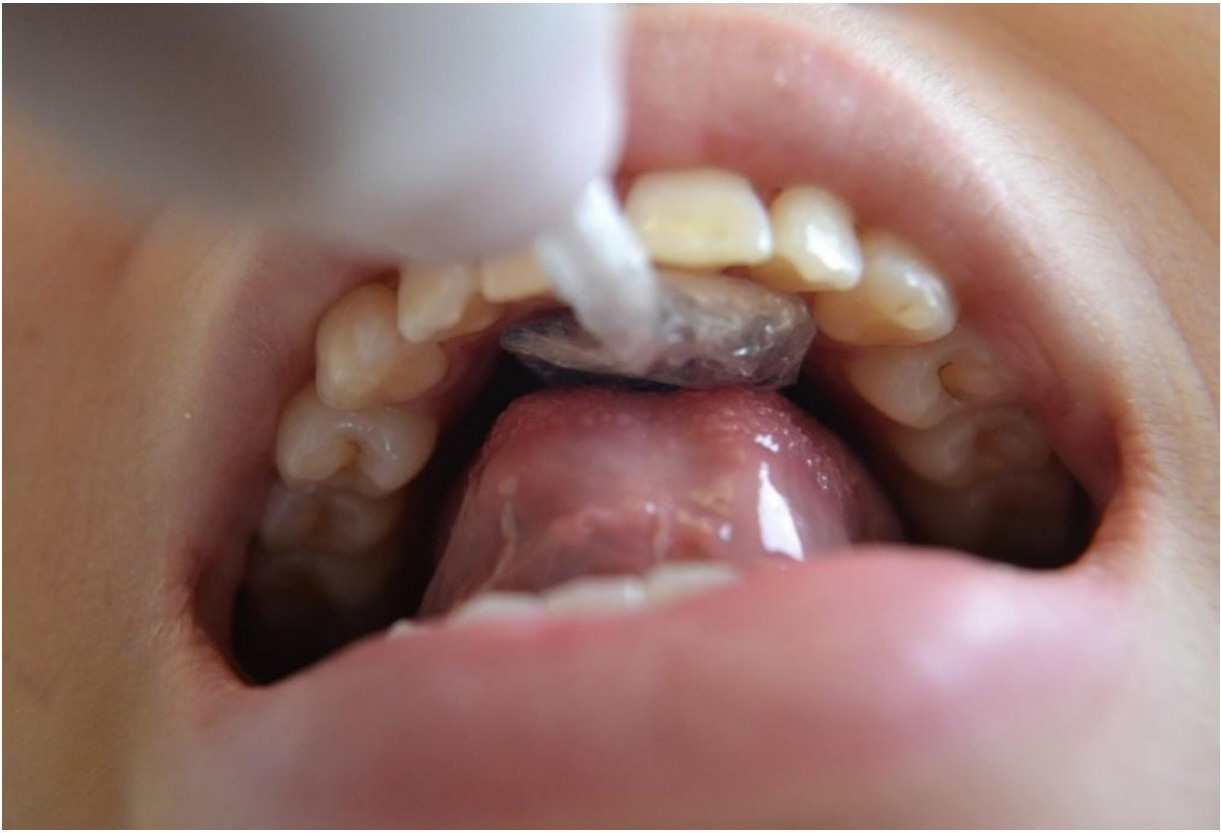
Anterior tongue pressure measurement

The participants were placed in a seated position with their backs and feet supported. Tongue pressure, lip pressure and tongue endurance were measured by the Iowa Oral Performance Instrument (IOPI) (IOPI Medical LLC; Carnation, Washington, USA), which contains a light mode display and an air-filled bulb. To measure anterior tongue pressure, the bulb was positioned on the center of the tongue, immediately behind the central incisors^(24)^ (Figure 1). The examiner held the bulb stem at a point immediately anterior to the participant’s central incisors to ensure consistent positioning of the bulb. The participants’ mandibles were not restrained. The participants were asked to raise their tongues and squeeze the bulb against the palate as hard as they could for approximately 2 seconds. The examiner then removed the bulb from the participant’s mouth and attempted two other measurements in the same anterior position, in a total of three measurements.

**Figure 2:**
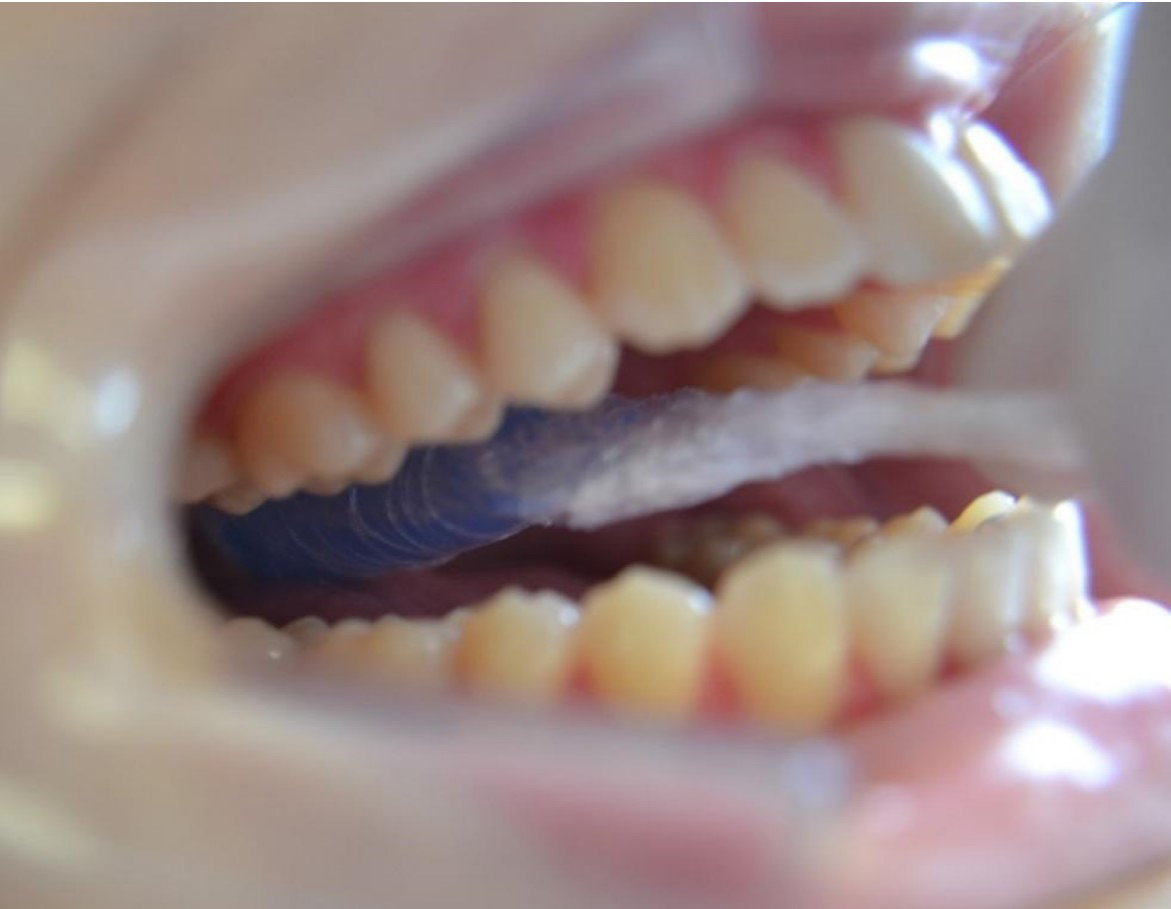
Posterior tongue pressure measurement

To measure posterior tongue pressure, the bulb was positioned on the tongue in a posterior position (anterior limit of the bulb parallel to the beginning of the first molars)^(14)^ (Figure 2), and the participants were asked to raise their tongues and squeeze the bulb against the palate as hard as they could for approximately 2 seconds three times. The order of measurements (anterior tongue pressure vs posterior tongue pressure) was random among the participants.

Tongue endurance was measured with the IOPI bulb in an anterior position on the tongue, measuring the time that the individual was able to maintain 50% of his/her maximum pressure, with monitoring by the equipment light. This measure was taken only one time to avoid the interference of fatigue.

**Figure 3:**
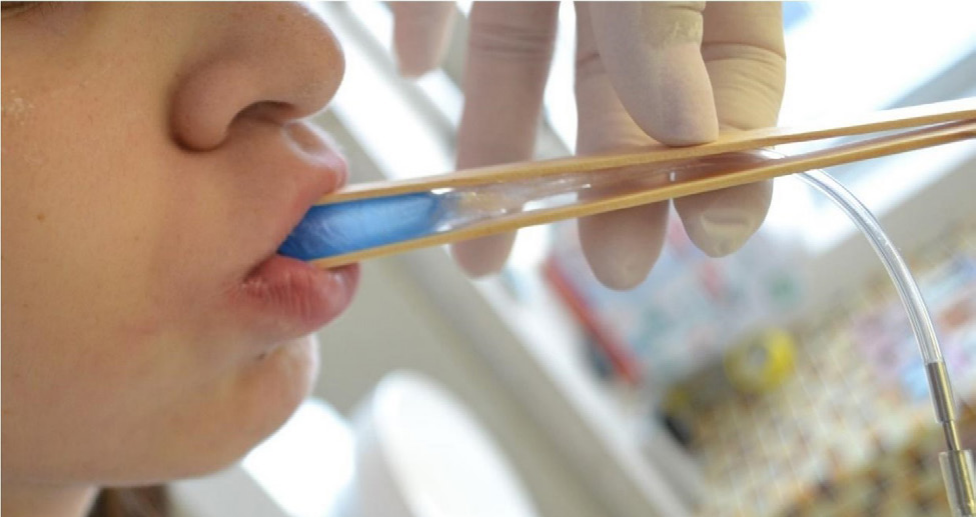
Lips pressure measurement

To measure the pressure of the lips, the bulb was positioned between two wooden spatulas (tongue depressors)^(6,25)^ (Figure 3), the spatulas were positioned between the midline of the lips, and the participant was instructed to place their teeth together and protrude the lips slightly after the blades were positioned, preventing them of applying pressure to the wooden spatulas using their jaw muscles. Then the participants were instructed to press it with the maximum force of the lips, three times. The placement of the bulb between spatulas, during measurements of lips force in protrusion task, is recommended by some authors as it allows a more uniform distribution of the labial force in the bulb^(25)^.

For each of these situations, a 30-second rest period was allowed between measurements. For anterior and posterior tongue pressure and for lips pressure, the highest value among the three measurements was considered.

### Statistical analysis

Descriptive analysis of the categorical variables (sex, type of malocclusion, and facial type) was carried out using the frequency analysis, and the descriptive analysis of continuous variables (age, anterior and posterior tongue pressure, lips pressure, and endurance) was performed using measures of central tendency and variability. The Kolmogorov-Smirnov test was used to assess the distribution of continuous variables. The compatibility of distribution of sex and facial type between Class I, Class II and Class III groups was assessed by chi-square test for multiple comparisons and the compatibility of age between the three malocclusion groups was performed by Kruskal Wallis test. Similarly, the compatibility of sex distribution between Horizontal, Mesofacial and Vertical groups was assessed by chi-square test for multiple comparisons and the compatibility of age between the three facial types groups was performed by Kruskal Wallis test. The comparison of tongue pressure, lips pressure, and tongue endurance between malocclusion types, between facial types, between malocclusion types for each facial type subgroup, and between sexes for each malocclusion type was performed by ANOVA tests with Bonferroni correction for variables with normal distribution and Kruskal Wallis with Dunn post hoc test for variables that did not follow a normal distribution. The adopted significance level was 5%. Analyses were performed using SPSS v23.0 software (IBM, New York, USA).

## RESULTS

The study included 55 individuals, with a minimum age of 18 years and a maximum age of 55 years (mean = 27.4 years, standard deviation = 9.0 years), 53% (n = 29) were male. Of the participants included, 51% (n = 28) had Class I malocclusion, 25% (n = 14) had Class II malocclusion, and 24% (n = 13) had Class III malocclusion. The horizontal facial type was found in 36% (n = 20) of the participants, mesofacial type in 35% (n = 19), and vertical facial type in 29% (n = 16) of the participants.

Table 1 presents the sample distribution according to the Angle classification and indicates that the groups were homogeneous in terms of sex, facial type, and age.

**Table 1:** Comparison of sex, facial type, and age between participants with different malocclusions (n=55)

Variables		Angle classification			p-value
		Class I	Class II	Class III	
Sex	Male	15	6	8	0.619*
	Female	13	8	5	
Facial type	Horizontal	13	4	3	0.349*
	Mesofacial	10	5	4	
	Vertical	5	5	6	
Age	Mean	26.6	28.2	28.3	0.810**
	Median	23.0	26.5	25.0	
	Standard Deviation	8.3	7.9	11.7	
	Minimum	18.0	18.0	18.0	
	Maximum	46.0	41.0	55.0	

* Chi-square test of multiple comparisons;

** Kruskal Wallis Test

Table 2 shows the sample distribution according to the facial type and indicates that the groups were homogeneous in terms of sex and age.

**Table 2:** Distribution of participants according to sex and age in different facial types (n=55)

Variables		Facial type			p-value
		Horizontal	Mesofacial	Vertical	
Sex	Male	8	11	10	0.347*
	Female	12	6	8	
Age	Mean	29.4	26.2	26.4	0.620**
	Median	27.5	25.0	23.0	
	Standard Deviation	10.5	8.0	8.1	
	Minimum	18.0	18.0	18.0	
	Maximum	55.0	46.0	46.0	

* Chi-square test of multiple comparisons;

** Kruskal Wallis Test

There was no statistically significant difference when comparing the malocclusion groups regarding tongue pressure, lip pressure, and tongue endurance (Table 3).

**Table 3:** Comparison of tongue pressure, lip pressure, and tongue endurance between malocclusion types

Variables		Class I	Class II	Class III	p-value
Anterior maximum tongue pressure (kPa)	Mean	56.1	57.0	58.1	0.881^b^
	Median	57.5	56.5	54.0	
	Standard Deviation	11.1	11.1	16.1	
	Minimum	29.0	39.0	36.0	
	Maximum	72.0	72.0	83.0	
Posterior maximum tongue pressure (kPa)	Mean	53.7	54.1	54.5	0.985^b^
	Median	57.5	54.5	58.0	
	Standard Deviation	14.7	14.0	15.4	
	Minimum	18.0	29.0	33.0	
	Maximum	77.0	75.0	78.0	
Tongue endurance (s)	Mean	46.4	54.1	51.3	0.950^a^
	Median	41.5	39.0	41.0	
	Standard Deviation	26.1	36.0	28.4	
	Minimum	17.0	18.0	18.0	
	Maximum	123.0	140.0	104.0	
Maximum lip pressure (kPa)	Mean	14.8	13.4	15.4	0.496^b^
	Median	14.0	14.0	15.0	
	Standard Deviation	4.8	3.4	4.8	
	Minimum	6.0	7.0	9.0	
	Maximum	30.0	19.0	27.0	

a Kruskal Wallis Test;

b Anova

There was a statistically significant difference in maximum posterior tongue pressure between the mesofacial and vertical types (Table 4).

**Table 4:** Comparison of tongue pressure, lip pressure, and tongue endurance between facial types

Variables		Horizontal	Mesofacial	Vertical	p-value
Anterior maximum tongue pressure (kPa)	Mean	58.3	60.2	50.8	0.057^b^
	Median	59.0	60.0	49.0	
	Standard Deviation	10.1	11.9	13.5	
	Minimum	36.0	36.0	29.0	
	Maximum	71.0	80.0	83.0	
Posterior maximum tongue pressure (kPa)	Mean	54.2	60.4	46.2	0.012^b^* Meso≠ Vertical (p=0.009c)
	Median	56.5	62.0	45.5	
	Standard Deviation	12.9	14.0	13.6	
	Minimum	18.0	26.0	28.0	
	Maximum	74.0	78.0	67.0	
Tongue endurance (s)	Mean	54.8	46.4	46.6	0.950^a^
	Median	42.0	41.0	36.0	
	Standard Deviation	34.5	19.2	32.1	
	Minimum	17.0	20.0	18.0	
	Maximum	123.0	86.0	140.0	
Maximum lip pressure (kPa)	Mean	14.8	14.4	14.6	0.957^b^
	Median	14.0	14.0	14.0	
	Standard Deviation	4.8	3.9	5.0	
	Minimum	9.0	6.0	7.0	
	Maximum	30.0	24.0	27.0	

a Kruskal Wallis Test;

b Anova;^c^Bonferroni

* p<0.05

**Caption:** Meso = mesofacial; Dolicho = dolichofacial.

Table 5 shows the comparison of tongue pressure, lip pressure, and tongue endurance between facial types in individuals with Class I, II, and III malocclusions. There were no statistically significant differences in the analyses performed.

**Table 5:** Comparison of tongue pressure, lip pressure, and tongue endurance between facial types, in subjects with Class I, II, and III malocclusion

Variables		Class I				Class II				Class III			
		Horizontal (n=13)	Meso (n=10)	Vertical (n=5)	p-value	Horizontal (n=4)	Meso (n=5)	Vertical (n=5)	p-value	Horizontal (n=3)	Meso (n=4)	Vertical (n=6)	p-value
Anterior maximum tongue pressure (kPa)	Mean	58.2	57.6	47.4	0.156^a^	61.5	57.4	53.0	0.554^a^	54.7	70.2	51.8	0.196^a^
	Median	59.0	59.5	49.0		62.5	54.0	54.0		64.0	73.5	49.0	
	SD	10.0	10.7	12.6		6.1	12.6	13.0		16.2	11.3	16.3	
	Minimum	39.0	36.0	29.0		54.0	43.0	39.0		36.0	54.0	36.0	
	Maximum	71.0	72.0	63.0		67.0	72.0	69.0		64.0	80.0	83.0	
Posterior maximum tongue pressure (kPa)	Mean	54.2	58.4	43.0	0.159^b^	54.2	60.0	48.2	0.445^b^	54.0	66.0	47.2	0.168^b^
	Median	57.0	59.0	43.0		52.5	64.0	55.0		61.0	68.0	45.5	
	SD	14.2	13.9	14.8		4.6	17.3	15.3		18.5	11.7	13.4	
	Minimum	18.0	26.0	28.0		51.0	33.0	29.0		33.0	50.0	33.0	
	Maximum	74.0	77.0	65.0		61.0	75.0	64.0		68.0	78.0	67.0	
Tongue endurance (s)	Mean	50.0	47.0	36.0	0.611^a^	61.5	47.2	55.0	0.858^a^	67.0	44.0	48.3	0.577^a^
	Median	38.0	48.0	36.0		63.5	32.0	36.0		78.0	38.5	40.5	
	SD	33.9	18.3	15.0		36.8	26.2	50.0		43.5	16.5	28.5	
	Minimum	17.0	20.0	21.0		20.0	26.0	18.0		19.0	31.0	18.0	
	Maximum	123.0	86.0	52.0		99.0	84.0	140.0		104.0	68.0	92.0	
Maximum lip pressure (kPa)	Mean	15.4	14.1	14.8	0.826^b^	13.2	13.6	13.4	0.989^b^	14.3	16.0	15.5	0.915^b^
	Median	14.0	13.5	17.0		13.5	14.0	15.0		15.0	17.0	13.0	
	SD	5.6	4.5	3.8		3.9	3.51	3.8		2.1	3.6	6.7	
	Minimum	9.0	6.0	9.0		9.0	10.0	7.0		12.0	11.0	9.0	
	Maximum	30.0	24.0	18.0		17.0	19.0	16.0		16.0	19.0	27.0	

a Kruskal Wallis Test;

b Anova

**Caption:** SD = Standard Deviation; Meso = mesofacial

Table 6 shows the comparison of tongue pressure, lip pressure, and tongue endurance between sexes in individuals with Class I, II, and III malocclusions. There were no statistically significant differences in the analyses performed.

**Table 6:** Comparison of tongue pressure, lip pressure, and tongue endurance between sex, in subjects with Class I, II, and III malocclusion

Variables		Class I			Class II			Class III		
		Male (n=15)	Female (n=13)	p-value*	Male (n=6)	Female (n=8)	p-value*	Male (n=8)	Female (n=5)	p-value*
Anterior maximum tongue pressure (kPa)	Mean	56.6	55.4	0.662	54.0	59.2	0.477	58.4	57.8	0.884
	Median	59	54		51.5	60.5		53	64	
	SD	11.8	10.7		13.9	8.7		16.2	17.7	
	Minimum	29	36		39	43		36	36	
	Maximum	71	72		72	69		83	75	
Posterior maximum tongue pressure (kPa)	Mean	55.7	51.5	0.596	53.7	54.5	0.897	52.6	57.6	0.660
	Median	58	56		54.5	55.5		48.5	61	
	SD	11.8	17.7		19.1	10.1		16.6	14.4	
	Minimum	28	18		29	33		33	33	
	Maximum	73	77		75	64		78	69	
Tongue endurance (s)	Mean	51.6	40.5	**0.062**	53.5	54.6	0.698	44.0	63,0	0.2134
	Median	45	30		34	46.5		33.5	68	
	SD	24.5	27.6		45.4	30.6		27.5	28.6	
	Minimum	21	17		18	20		18	31	
	Maximum	123	122		140	99		92	104	
Maximum lip pressure (kPa)	Mean	14.9	14.7	**0.853**	13.2	13.6	0.698	16.1	14.2	0.6605
	Median	14	14		13.5	15		14.5	15	
	SD	5.0	4.7		4.0	3.1		5.6	3.4	
	Minimum	9	6		7	9		11	9	
	Maximum	30	24		19	17		27	18	

* Kruskal Wallis Test

**Caption:** SD = Standard Deviation

## DISCUSSION

No statistically significant difference was observed in maximum anterior and posterior tongue pressure, lip pressure, or tongue endurance in the different malocclusion types. Few studies investigating orofacial strength in individuals with malocclusion were detected in the literature and they were accomplished mostly with participants with skeletal deformities. Silva et al.^(11)^ found no significant differences in tongue strength between adults with dentofacial deformities (Class II and Class III) and the controls (Class I) while Menezes et al.^(14)^ found lower values ​​of tongue pressure and endurance in skeletal Class II women than in the literature reference values. The authors^(14)^ did not evaluate Class I subjects but used literature values as a reference. The results of these studies^(11,14)^, however, should be interpreted with caution, as their participants, unlike those in this research, had skeletal malocclusion, being orthognathic surgery candidates.

Kuwajima et al.^(16)^ did not found difference between maximum tongue pressure in individuals with different type of dental malocclusion. Lambrechts et al.^(10)^ did not find a relationship between tongue pressure and malocclusion classification either, but lip pressure was lower in Class II division 1 than in Class I individuals. In the present study, we did not separate Class II participants according to divisions and subdivisions. However, it is important to note that the study of Lambrechts, et al. included a sample of adults and children^(10)^. Another study found significantly lower tongue pressure in skeletal Class II children compared to Class I and Class III groups^(13)^. Likely, the strength and endurance of the lips and tongue in subjects with malocclusion undergo adaptations in the course of craniofacial growth and development so that it can perform its functions^(11)^. Thus, although the literature indicates a decrease in strength in children with dental or/and skeletal changes, these differences probably disappear as these subjects approach adulthood.

On the other hand, a statistically significant difference was found when comparing the maximum posterior tongue pressure between mesofacial and vertical individuals. Other authors reported a higher occurrence of changes in tongue posture, lip posture, speech, and respiratory function in vertical individuals^(18,26)^. However, we found no study that investigated the maximum tongue or lip force in different facial types. One study^(27)^ investigated tongue pressure during swallowing in five subjects of each facial type and found higher values of tongue pressure during swallowing in vertical subjects followed by mesofacial and horizontal, which contradicts the findings of the present study. However, the behavior of tongue strength during maximal contraction is different from its behavior during orofacial functions. This does not require the maximal activity of the musculature. An explanation for the lower posterior maximum tongue pressure in vertical participants may be related to the low habitual posture of the tongue in the oral cavity, which can affect the tone. It is possible that during swallowing, these individuals increase the effort made by the tongue, using more of their reserve strength than individuals with other facial types.

The posterior part of the tongue has a predominance of type I muscle fibers^(28)^. This type of muscle fiber is slower in contraction, more resistant to fatigue due to the greater capacity to produce adenosine triphosphate by aerobic metabolism, but it has a lower capacity to generate force compared to type II fibers, which are predominant in the anterior portion of the tongue^(28)^. It is assumed that this differentiation between the portions of the tongue, with type I fibers in the posterior part, makes this region more susceptible to loss of tone caused by a lowered habitual position. Another possible factor is the greater concentration of muscle tissue in this region, when compared to the anterior part^(29)^, making it more susceptible to the loss of strength in dolichocephalic individuals. The anterior region of the tongue has a higher concentration of connective tissue than the middle and posterior regions^(29)^, which may have mitigated the loss of strength due to the low posture.

The groups were homogeneous in terms of age, sex, and facial type, which is important as some studies have indicated that tongue strength is different between sexes^(10,30)^, and also influenced by age, being lower in children and the elderly when compared to young adults^(31)^.

When the values of the tongue and lip pressures were compared between facial types within each group, there was no significant difference, which probably occurred due to the reduction of the sample size, since the groups when stratified had fewer participants in each subgroup. The same may have happened when those pressures were compared between sexes in individuals with Class I, II, and III malocclusions, as the literature points out in a direction of men generally showing higher tongue^(10,30)^ and lip strength^(6)^.

In the analysis of systematic error, no variable showed a statistically significant difference between the two measurements. In the analysis of the casual error, four of the six variables analyzed showed values ​​above those recommended in the evaluation of the casual error: facial axis angle, facial depth, mandibular plane angle, and mandibular arch. Considering the mean values ​​of the angles, except for the mandibular arch, the other variables showed casual error above the ideal presented small magnitude deviations, without affecting the facial type. These values ​​are within the standard deviations recommended by Ricketts^(32)^ analysis. The mandibular arch, however, revealed considerable casual errors. However, its contribution from this angle in the calculation of the VERT index is small since the variable is divided by its standard deviation and is considered together with the other four variables in the definition of the facial type. In this way, the results of the intra-examiner error demonstrated that there was acceptable precision in marking the cephalometric points and in the measurement of the variables, guaranteeing the reproducibility and reliability of the results obtained in this research.

High standard deviations were observed, which is explained by the individual variability inherent to this type of measurement, which was also verified by other authors^(24,30)^. Furthermore, the assessment of the pressure of orofacial structures is not an objective assessment, but a semi-objective one, as it depends on the individual's understanding and willingness to exert maximum pressure on the instrument, which increases the variability of inter-subject measurements.

The hypothesis that tongue pressure, tongue endurance and lips pressure would be reduced in Class II and Class III individuals compared to Class I individuals were not confirmed. We believe that these individuals can develop adaptive mechanisms for habitual position and lingual functions, which maintain the strength. The second hypothesis, that vertical individuals would have lower values compared to horizontal individuals, was confirmed for one of the variables – posterior tongue pressure. Probably the lower position the tongue assumes inside the mouth in vertical individuals is responsible to diminish its tone, being more critical for this region of the tongue because of its anatomical constitution.

This study, by evaluating the pressure of the tongue and lips of adults with different types of occlusions, adds evidence to the literature, showing that malocclusion, based on the Angle Classification, does not seem to influence the maximum pressure of the tongue and lips in adulthood. However, the vertical facial type deserves more attention, as it determines a decrease in posterior tongue strength. Further investigations should be carried out with other age groups, especially the elderly, in order to verify whether old individuals with vertical facial type are more susceptible to functional swallowing changes due to loss of force in the posterior part of the tongue.

A limitation of this study is the small sample size, especially in the subgroups of occlusions and facial types. There is a difficulty in finding individuals who met all the inclusion criteria, especially the presence of all first molars in adulthood. It is important to note that the total number of participants predicted in the sample calculation was reached. Suggestions for further research include increasing the sample size, including other age groups, and investigating tongue and lip pressures during swallowing.

## CONCLUSION

There was no difference in tongue pressure, tongue endurance, or lip pressure between individuals with different malocclusion classifications. Individuals with vertical facial type had lower maximum anterior and posterior tongue pressures than mesofacial individuals.
